# Activation of Human Mucosal-Associated Invariant T Cells Induces CD40L-Dependent Maturation of Monocyte-Derived and Primary Dendritic Cells

**DOI:** 10.4049/jimmunol.1700615

**Published:** 2017-09-06

**Authors:** Mariolina Salio, Olivier Gasser, Claudia Gonzalez-Lopez, Anne Martens, Natacha Veerapen, Uzi Gileadi, Jacob G. Verter, Giorgio Napolitani, Regan Anderson, Gavin Painter, Gurdyal S. Besra, Ian F. Hermans, Vincenzo Cerundolo

**Affiliations:** * Medical Research Council Human Immunology Unit, Weatherall Institute of Molecular Medicine, University of Oxford, Oxford OX3 9DS, United Kingdom;; † Malaghan Institute of Medical Research, School of Biological Sciences, Victoria University of Wellington, Wellington 6242, New Zealand;; ‡ School of Biosciences, University of Birmingham, Birmingham B11 2TT, United Kingdom;; § The Ferrier Research Institute, Victoria University of Wellington, Lower Hutt 5046, New Zealand; and; ¶ Maurice Wilkins Centre for Molecular Biodiscovery, Auckland 1042, New Zealand

## Abstract

Mucosal-associated invariant T (MAIT) cells are innate T cells that recognize intermediates of the vitamin B2 biosynthetic pathway presented by the monomorphic MR1 molecule. It remains unclear whether, in addition to their cytolytic activity that is important in antimicrobial defense, MAIT cells have immune-modulatory functions that could enhance dendritic cell (DC) maturation. In this study, we investigated the molecular mechanisms dictating the interactions between human MAIT cells and DCs and demonstrate that human MAIT cells mature monocyte-derived and primary DCs in an MR1- and CD40L-dependent manner. Furthermore, we show that MAIT cell–derived signals synergize with microbial stimuli to induce secretion of bioactive IL-12 by DCs. Activation of human MAIT cells in whole blood leads to MR1- and cytokine-dependent NK cell transactivation. Our results underscore an important property of MAIT cells, which can be of translational relevance to rapidly orchestrate adaptive immunity through DC maturation.

## Introduction

The innate and adaptive arms of the immune system require tight regulation for the induction of protective immunity against pathogens and prevention of autoimmunity. In addition to conventional peptide-specific MHC-restricted T cells, an expanding population of “in-betweeners,” or unconventional cells, exists (1). These cells bear adaptive rearranged TCRs, yet with limited diversity, they display innate-like behavior, a memory phenotype, can be rapidly activated, and orchestrate adaptive immunity through dendritic cell (DC) maturation (1). Unconventional T cell populations include CD1-restricted T cells, γδ T cells with various restriction, and MHC class Ib–restricted T cells (2). MR1-restricted (mucosal-associated invariant T) MAIT cells are a recently described addition to the unconventional T cell family, recognizing unstable adducts derived from a precursor of the riboflavin (vitamin B2) pathway, which is present in a number of bacterial and fungal species (3). Although the details of MR1-restricted Ag presentation are being unraveled (4), a number of questions about the biology of these cells remain unanswered. MR1-deficient mice, which lack MAIT cells, are more susceptible to some bacterial infections, such as *Klebsiella*, *Mycobacterium bovis* bacillus Calmette-Guérin, and *Francisella tularensis*, suggesting an important role for MAIT cells in antibacterial immunity (reviewed in Ref. 5). Recently, in a mouse model of *F. tularensis* infection, it has been shown that MAIT cells promote GM-CSF–dependent, but MR1-independent, differentiation of inflammatory monocytes into monocyte-derived DCs, influencing early activation and recruitment of T cells (6). These results suggest that cross-talk between MAIT cells and myeloid cells may be important to shape Ag-specific adaptive immunity, as previously observed for CD1d-restricted invariant NKT (iNKT) cells. Therefore, we investigated the molecular mechanisms dictating the outcome of interactions between human MAIT cells and DCs and demonstrate the ability of human MAIT cells to mature monocyte-derived and primary DCs.

## Materials and Methods

### Medium and reagents

The complete medium (CM) used throughout was RPMI 1640 (Life Technologies) for DCs and IMDM (Life Technologies) for human MAIT cells. CM was supplemented with 2 mM l-glutamine, 1% nonessential amino acids, 1% sodium pyruvate, 1% Pen/Strep, 5 × 10^−5^ 2-ME (all from Life Technologies) and serum: 10% FCS or 5% Human AB Serum (both from Sigma) for MAIT cells. MAIT cell medium was supplemented with 1000 U/ml recombinant human IL-2, which was produced in our Oxford laboratory as described (7).

Ultrapure LPS and R848 were purchased from InvivoGen. Methylglyoxal (MG; 40% in water) was from Sigma. DH5α *Escherichia coli* bacteria (Invitrogen) were grown overnight to stationary phase in Luria broth, and the supernatant was centrifuged and sterile filtered before use as an enriched source of MAIT ligands (30, 10, or 3 μl in a 200-μl assay).

5-Amino-6-d-ribitylaminouracil (5-A-RU) was synthesized as follows. 6-Ribitylaminouracil was synthesized from d-ribose, as previously reported (8), and purified by ion-exchange chromatography (9). Nitrosation at the five position was carried out with a slight modification of the procedure described in the literature (8), using sodium nitrite and barium acetate in place of barium nitrite. The product, 5-nitroso-6-ribitylaminouracil, was purified by ion-exchange chromatography (9). Reduction of the nitroso group using sodium dithionite gave 5-A-RU (3), which was used without further purification. The product was analyzed by liquid chromatography–mass spectrometry on an Agilent 1260 HPLC system equipped with an Agilent 6130 single quadrupole mass spectroscopic detector. For the chromatographic conditions a Phenomenex Synergi Fusion-RP column (2.5 μm, 100 Å, 50 × 3 mm) was used, with elution in isocratic 10 mM aqueous ammonium acetate (0.5 ml/min, 30°C). 5-A-RU was detected by UV (214, 254 nm) and mass spectrometry (electrospray ionization positive, m/z = 277 [positive ionization mode]; electrospray ionization negative, m/z = 275 [negative ionization mode]) at 1.05 min.

### Generation of MAIT cells and DCs

Blood was purchased from the UK National Blood Service. Human MAIT cells were isolated by sorting CD2 MACS-enriched leukocytes with CD161 and Vα7.2 Abs (BioLegend). MAIT cells were grown for a few weeks in CM supplemented with IL-2. Control CD8^+^ CD161^+^ and CD8^+^ CD161^+^ cells were sorted from CD2-enriched leukocytes at the same time as MAIT cells and simultaneously cultured.

DCs were differentiated by culturing CD14 MACS-purified monocytes in CM supplemented with human GM-CSF (50 ng/ml) and human IL-4 (1000 U/ml, both from PeproTech).

### Whole-blood assays

Freshly drawn blood was distributed in 5-ml polypropylene conical tubes (BD Falcon). One milliliter of blood was activated with 5-A-RU and MG (1 μg/ml and 100 μM, respectively), in the presence of 30 μg/ml isotype-control, anti-MR1 (clone 26.5), or anti-CD40L (clone 24.31) Abs. Anti–IL-18R1 (clone H44) was used for NK cell experiments. LPS was used at 1 μg/ml. After 2 h of stimulation, protein transport inhibitor containing Brefeldin A (BD) was added, and stimulation was continued for an additional 16 h. Cells were surface stained in Brilliant Violet buffer (BD), fixed and permeabilized (eBioscience), and stained for intracellular cytokines. The following Abs were used throughout: BV785 CD123 (7G3; BioLegend), BUV737 IFN-γ (4S.B3), BUV395 TNF-α (MAb11), BUV661 CD3 (UCHT1), BUV563 CD56 (NCAM16.2; all from BD), CD33 PE or allophycocyanin (P67.6), PE/Dazzle CD137 (VI C-7), PE IL-12p40 (C11.5), allophycocyanin or BV510 CD161 (HP-3G10), BV605 Vα7.2 (3C10), BV605 CD14 (M5E2), BV510 CD19 (HIB19; all from BioLegend), FITC CD40L (TRAP1; BD), Alexa Fluor 700 CD11b (M1/70), BV650 CD16 (3G8), PECy7 CD1c (L161), allophycocyanin Cy7 HLA DR (L243), allophycocyanin BDCA2 (201A), BV711 CD40 (5C3), BV421 CD80 (2D10; all from BioLegend), BB515 CD86 (FUN-1; BD), PerCPCy5.5 CD141 (M80), BV785 CD38 (HIT2), FITC CD28 (28.2), PE/Dazzle CD27 (1G3A10), PECy7 NKG2D (1D11; all from BioLegend), PE NKG2A (REA110; Miltenyi Biotec), and PerCPCy5.5 KIR2DL1 (HP MA4; BioLegend). Samples were acquired on an X50 BD symphony machine and analyzed with FlowJo 10. Viability was assessed with LIVE/DEAD Aqua staining, according to the manufacturer’s instructions (Invitrogen).

### Stimulation assays

DCs were plated at 50,000 cells per well in 96-well flat-bottom plates in CM and incubated with MAIT cells (20,000 cells per well, in triplicate) in the presence or absence of different concentrations of 5-A-RU/MG, bacterial supernatant, LPS, or R848. Depending on availability, autologous and/or allogeneic MAIT cells were used in different experiments because MR1 lacks polymorphism. For blocking experiments, DCs were incubated for 1 h with the ligands and then for 2 h with 30 μg/ml isotype controls, anti-MR1 (clone 26.5), anti-CD40L (clone 24.31), or anti–IL-12 (clone C8.6) Abs before the addition of MAIT cells. MAIT cell and DC activation was assessed by flow cytometry after 36 h with the following Abs: FITC CD83 (HB15e), allophycocyanin CD86 (FUN1), PE CD80 (L307.4; all from BD), PECy7 PDL1 (29E.2A3; BioLegend), and PE-CF594 CD25 (M-A251; BD). IFN-γ, IL-12p40, and IL-12p70 were also measured by ELISA (Abs from BD) on supernatants harvested after 36 h. We did not detect any IL-17 or IL-23 in our supernatants (ELISA Ab pairs from eBioscience, sensitivity 0.04 and 0.39 ng/ml, respectively).

### Transwell assay

Costar 24-well plates with a 0.4-μm pore size insert were used (catalog number 3470). A total of 100,000 DCs was plated in the bottom well in 800 μl of medium; in the top well, 60,000 DCs were plated with 50,000 allogeneic MAIT cells in 200 μl of medium. 5-A-RU was used at 100 ng/ml, with 100 μM MG and 30 μg/ml blocking Abs, as described above. As a positive control for maturation, 50 μl of supernatant of a 6-ml overnight culture of spun DH5α bacteria was added to the DCs. DCs were collected and stained after 36 h of culture.

### Simoa immunoassay

To detect very low levels of IL-12p70, supernatants were subjected to automatized ELISA analysis with the Simoa HD-1 Analyzer and Single Molecule Array (Simoa) technology (Quanterix), using the Simoa Human IL-12 p70 kit, following the recommendations of the manufacturer (10).

### Statistical analyses

Statistical analyses were performed with GraphPad Prism software, version 7. Comparisons were performed using the Wilcoxon matched-pairs signed-rank test and differences with *p* < 0.05 were deemed significant. In experiments with *n* = 2, a statistical test was not performed because of the low sample size.

## Results

### Activated human MAIT cells upregulate CD40L

**FIGURE 1.**
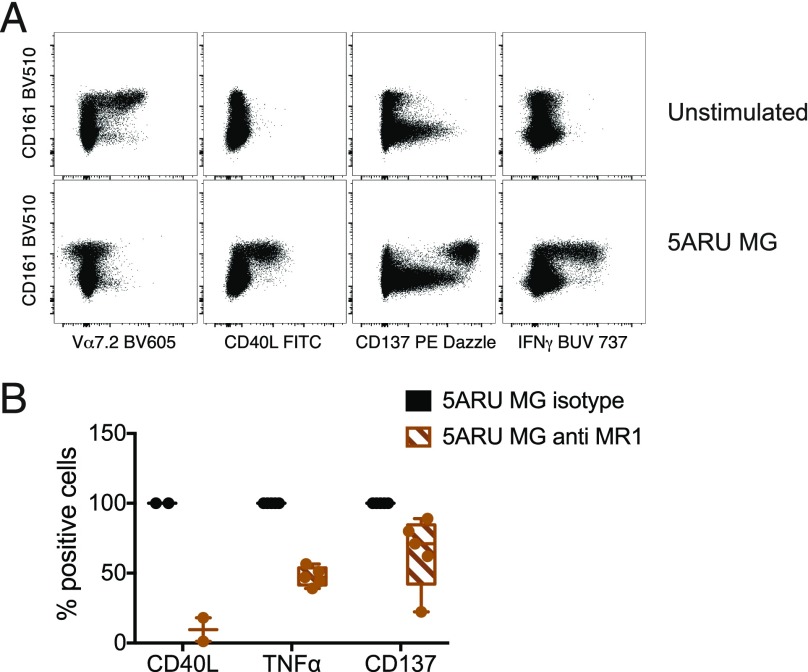
Human MAIT cell activation leads to MR1-dependent CD40L upregulation. Whole blood was incubated for 16 h with 5-A-RU and MG in the presence of protein transport inhibitors. (**A**) Expression of Vα7.2 TCR, CD40L, CD137, and IFN-γ was detected by intracellular staining and is depicted in the FACS dot plots, in parallel with CD161 expression. (**B**) Effect of MR1 blockade on CD40L (*n* = 2), TNF-α (*n* = 5), and CD137 (*n* = 5) expression in the intracellular assay performed as depicted in (A). Data are mean ± SD. For CD137-expressing cells, the ratio of mean fluorescence intensity with and without anti-MR1 is plotted, because the overall percentage of positive cells remains unchanged (see also Supplemental Fig. 4B). Statistical analysis was not performed for CD40L because of the small sample size. *p* = 0.06 for TNF-α and CD137, Wilcoxon signed-rank test.

We synthesized 5-A-RU, an early intermediate in bacterial riboflavin synthesis that can form simple adducts with cellular metabolites to provide MR1-binding MAIT cell agonists (11, 12). This compound induced potent dose-dependent and MR1-restricted activation of MAIT cell lines derived from healthy donors (Supplemental Fig. 1A). As previously described, MAIT cell activation was much stronger in the presence of exogenous MG, likely as a result of the formation of the potent adduct 5-OP-RU, which is known to be a potent agonist (Supplemental Fig. 1B) (11). We next tested 5-A-RU/MG on unfractionated MAIT cells in whole blood and identified MAIT cells by Vα7.2 and CD161 costaining (13). Upon MAIT cell activation, we observed strong Vα7.2 TCR downregulation that was accompanied by IFN-γ secretion and upregulation of the activation marker CD137, both of which were confined to CD161^bright^ cells (Fig. 1A). Interestingly, MAIT cell activation was accompanied by CD40L upregulation (Fig. 1, Supplemental Fig. 1C). In these cocultures, CD40L was specifically expressed on IFN-γ–secreting CD137^+^ CD161^bright^ cells (Fig. 1, Supplemental Fig. 1C). The specificity of MAIT cell activation was demonstrated with anti-MR1 Abs, which blocked CD40L expression and TNF-α secretion (Fig. 1B). However, anti-MR1 Abs only marginally reduced CD137 expression (Fig. 1B, Supplemental Fig. 4).

### Human MAIT cell lines induce CD40L-dependent DC maturation

**FIGURE 2.**
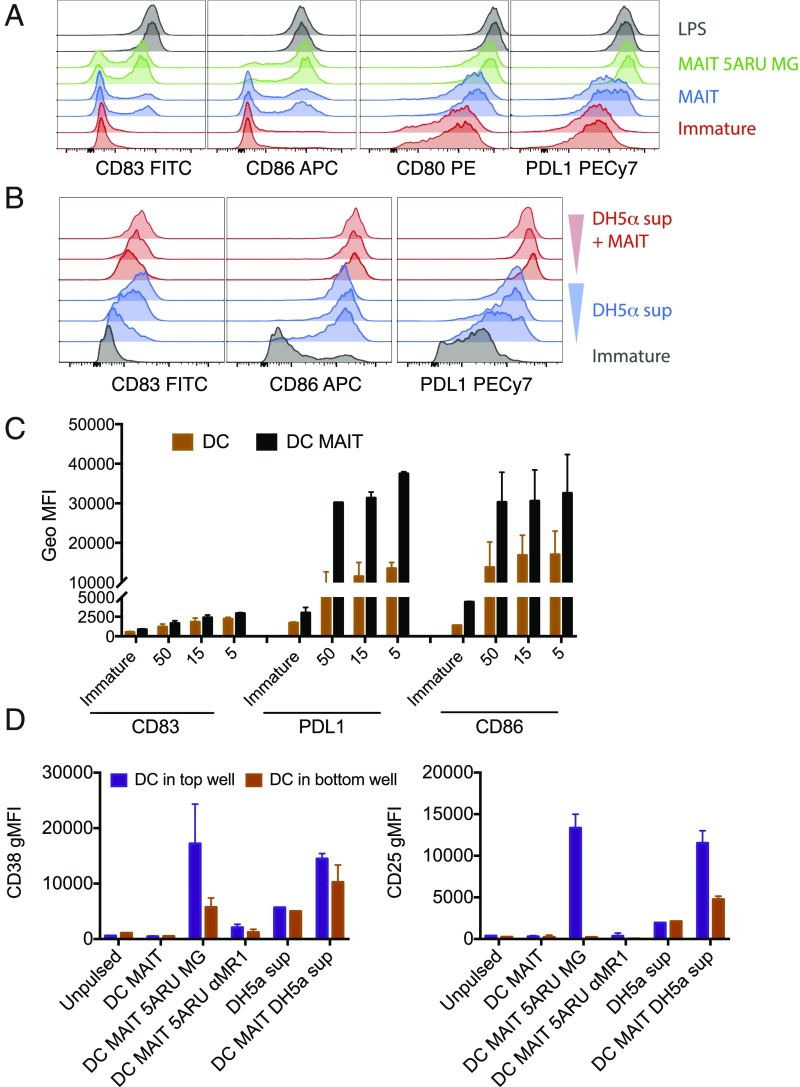
Human MAIT cells induce DC maturation. (**A**) Immature DCs (red graphs) were incubated with allogeneic MAIT cells in the presence (green histograms) or absence (blue graphs) of 5-A-RU/MG. LPS (gray graphs) was used as positive controls. Staining profile of duplicate wells is shown. (**B**) Immature DCs (gray graphs) were incubated with three concentrations of DH5-α supernatant in the presence (red graphs) or absence (blue graphs) of autologous MAIT cells. Depicted are expression levels of CD83, CD86, CD80, and PDL1 as detected by flow cytometry after 36 h. (**C**) One representative experiment of four; data from two donors (one autologous and one allogeneic to the DCs) are tabulated; 50, 15, and 5 on the *x*-axis refer to different amounts of DH5-α supernatant. (**D**) Expression of CD38 and CD25 (geometric mean ± SD of two donors) on DCs harvested from the top and bottom wells of a Transwell assay. Where indicated, allogeneic MAIT cells were added in the top wells, in the presence or absence of 5-A-RU/MG or supernatant of DH5-α– and anti-MR1–blocking Abs.

Ligation of CD40 on human and murine DCs triggers production of high levels of IL-12 and provides a source of help required for efficient priming of CTLs (14–16). In addition to CD4^+^ T cells, iNKT cells have been shown to be potent inducers of CD40-dependent DC maturation (17, 18). Therefore, we investigated whether MAIT cells could also trigger DC maturation and IL-12 secretion. When coincubated with monocyte-derived immature DCs in the absence of exogenous ligands, we observed that MAIT cells induced partial DC maturation, as defined by an increase in the expression of CD80, CD83, CD86, and PD-L1 (Fig. 2A, blue lines). DC maturation was clearly enhanced in the presence of the synthetic cognate ligand 5-A-RU/MG (Fig. 2A, green lines), almost reaching the levels observed with low-dose LPS (Fig. 2A, gray lines). Furthermore, MAIT cells enhanced maturation induced by supernatant of DH5α *E. coli* (Fig. 2B, 2C), which has been shown to contain vitamin B2–derived activating ligands (3, 19). This effect could be partially blocked with anti-MR1 Abs (Supplemental Fig. 1D). To further dissect the contribution of MR1 and soluble factors in dictating DC maturation, we performed Transwell experiments (Fig. 2D). DCs were plated in the top and bottom wells of a Transwell chamber, whereas MAIT cells and stimuli were added to the top well. We observed that 5-A-RU/MG–dependent DC maturation was primarily driven by cognate interactions, because we detected it mainly in the DCs harvested from the top wells. In addition, in both wells, DC maturation could be blocked by anti-MR1 (Fig. 2D). Finally, we observed that MAIT cells enhanced DC maturation induced by DH5α *E. coli* supernatants in both wells, suggesting a possible contribution of soluble factors in this specific setting (Fig. 2D). We excluded the possibility that DC maturation was due to endotoxin or other innate signaling contaminants because, in the absence of MAIT cells, 5-A-RU/MG did not induce DC maturation, even at a high concentration (150 μg/ml) (Supplemental Fig. 1E).

**FIGURE 3.**
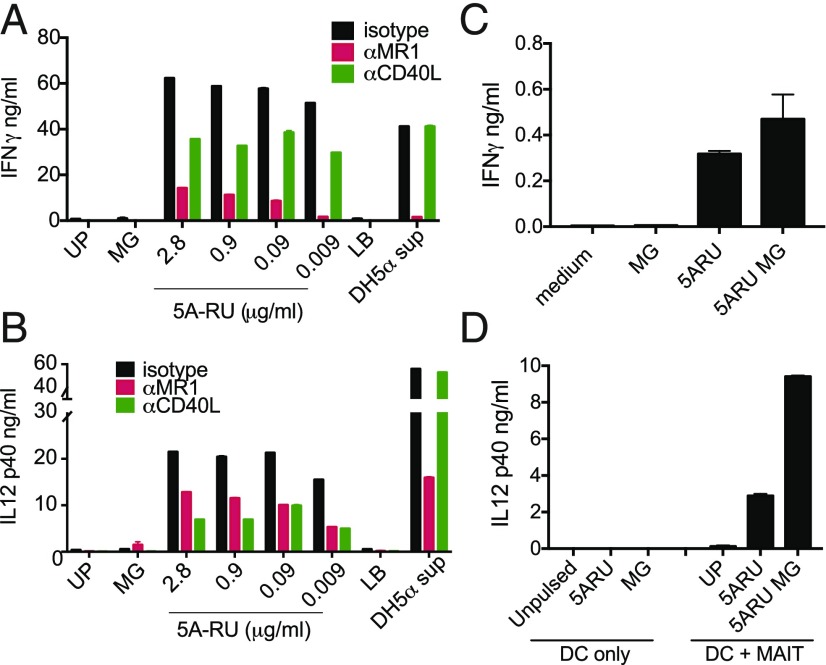
Human MAIT cells induce CD40L-dependent DC maturation. IFN-γ (**A**) and IL-12p40 (**B**) levels in the supernatant of MAIT-allogeneic DC cocultures pulsed with the indicated concentration of 5-A-RU and constant MG (50 μM) in the presence or absence of anti-MR1 or anti-CD40L blocking Abs. (**C**) IFN-γ levels in the supernatant of MAIT cells exposed to 5-A-RU and/or MG in the absence of allogeneic DCs. Note the difference in the *y*-axis scale. (**D**) IL-12p40 in the supernatant of DCs alone or DCs and allogeneic MAIT cell cocultures pulsed with 5-A-RU and/or MG. One experiment representative of three; data are mean ± SD.

We next investigated by ELISA whether cytokines were released into the supernatant when human MAIT cells were cocultured with 5-A-RU/MG–pulsed DCs. We observed dose-dependent release of IFN-γ and IL-12p40, which could largely be blocked with anti-MR1, likely reflecting release by MAIT cells and DCs, respectively (Fig. 3A, 3B). In keeping with the concept that MAIT cells can license DCs via CD40, IL-12 p40 production was blocked with anti-CD40L (Fig. 3B). Interestingly, anti-CD40L also reduced IFN-γ secretion by MAIT cells (Fig. 3A), which may be a consequence of the reduction of cytokines, such as IL-12, which could feed back on MAIT cells via highly expressed receptors (20). Because MR1 is ubiquitously expressed (21), we ruled out that MAIT cell activation was due to presentation of 5-A-RU/MG by neighboring MAIT cells (Fig. 3C) and also demonstrated that DCs did not release any IL-12 in the absence of MAIT cells (Fig. 3D). We did not detect any IL-23 or IL-17 in our coculture experiments (data not shown).

**FIGURE 4.**
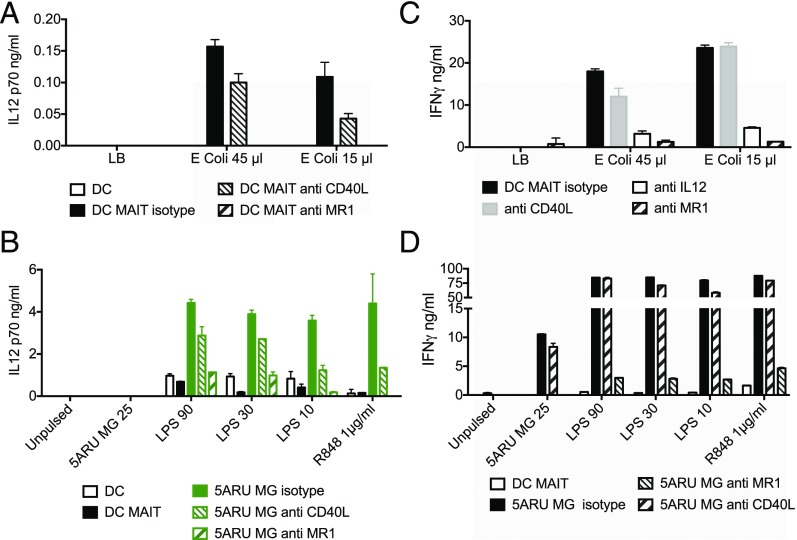
Synergy between human MAIT cell agonists and TLR agonists. (**A**) Bioactive IL-12p70 in the supernatant of DCs pulsed with *E. coli*upernatant in the presence or absence of allogeneic MAIT cells and blocking anti-CD40L or anti-MR1 Abs. (**B**) Bioactive IL-12p70 in the supernatant of DCs pulsed with the indicated concentrations of 5-A-RU/MG (ng/ml), LPS (ng/ml), or R848 (μg/ml) in the presence or absence of allogeneic MAIT cells and blocking anti-CD40L or anti-MR1 Abs. (**C**) IFN-γ levels in the supernatant of MAIT-allogeneic DC cocultures pulsed with *E. coli* supernatant in the presence or absence of blocking anti-CD40L, anti–IL-12, or anti-MR1 Abs. (**D**) IFN-γ levels in the supernatant of MAIT–allogeneic DC cocultures pulsed with the indicated concentrations of 5-A-RU/MG (ng/ml), LPS (ng/ml), or R848 (μg/ml) in the presence or absence of blocking anti-CD40L or anti-MR1 Abs. Data are from one experiment representative of four; data are mean ± SD. Two more donors are shown in Supplemental Fig. 2.

In the above coculture experiments, IL-12p40 was easily detected, whereas IL-12p70 was below the detection limit of our ELISA. Therefore, we investigated whether signals from activated MAIT cells could cooperate with stimuli via pathogen-derived pattern recognition receptors to induce release of the bioactive form of IL-12 by DCs. First, we observed that when DCs were incubated with supernatants of *E. coli* DH5α bacteria, they secreted IL-12p70 only in the presence of MAIT cells, and this could be completely abrogated by anti-MR1 Abs and partially abrogated by anti-CD40L Abs (Fig. 4A, Supplemental Fig. 2A). We next extended these results and demonstrated that, in the presence of low doses of 5-A-RU/MG, MAIT cells significantly enhanced IL-12p70 secretion induced by the TLR agonists LPS and R848 (Fig. 4B, Supplemental Fig. 2C, 2D). This effect was entirely MR1 dependent, with some contribution of CD40L at low concentrations of TLR ligands. In turn, we observed an IL-12 and MR1–dependent MAIT cell activation by *E. coli*–pulsed DCs (Fig. 4C, Supplemental Fig. 2B). In addition, at the concentrations tested, TLR ligands potently synergized with 5-A-RU/MG in inducing MR1-dependent MAIT cell activation (Fig. 4D, Supplemental Fig. 2E).

**FIGURE 5.**
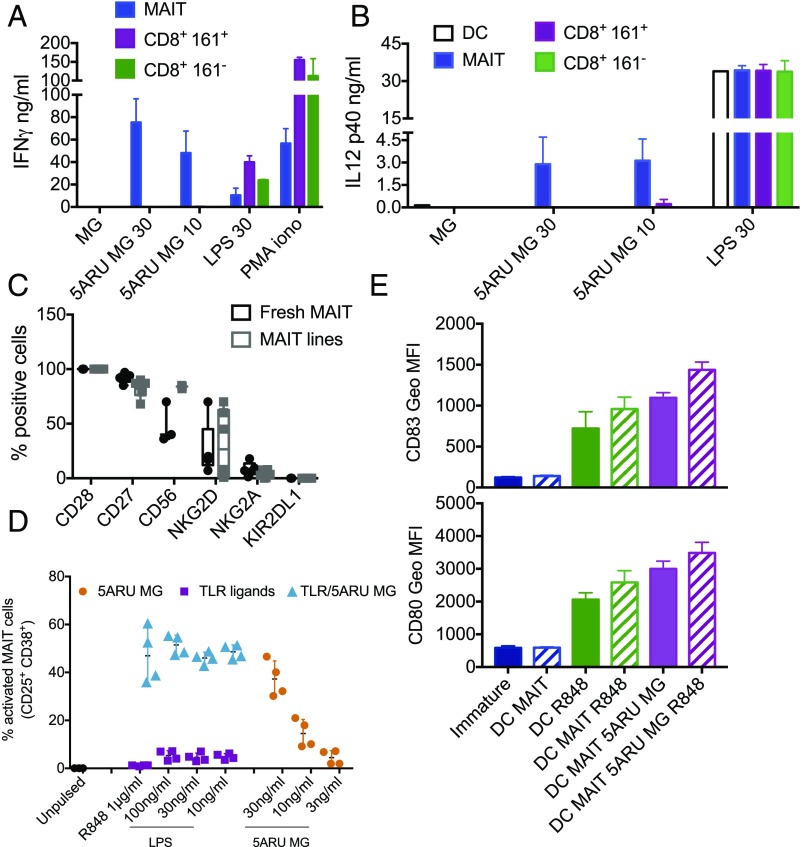
MAIT cells, but not CD8 T cells, induce 5-A-RU/MG–dependent DC maturation. IFN-γ (**A**) and IL-12 (**B**) secretion in cocultures of DCs and MAIT cells, CD8^+^ CD161^+^ cells, or CD8^+^ CD161^−^ cells, activated by the indicated concentration of 5-A-RU/MG or LPS. PMA and ionomycin was used as a control for T cell activation. Data are from two experiments, with one MAIT cell donor autologous to and one allogeneic to the DCs; values represent mean ± SD. (**C**–**E**) Freshly sorted MAIT cells induce DC maturation. (C) Phenotype of freshly sorted MAIT cells and MAIT lines. Plotted is the percentage of cells positive for each indicated marker, depicted as box-and-whisker plots, with all points indicated. *n* = 5 for fresh MAIT cells;*n* = 6 for MAIT lines (only three of each were tested for CD56). For two donors, fresh and cultured MAIT cells were tested. (D) Percentage of freshly sorted MAIT cells activated in response to DCs pulsed with the indicated concentration of 5-A-RU/MG, TLR ligands, or a combination of the two. (E) Expression of CD83 and CD80 (geometric mean ± SD) in DCs activated by freshly sorted MAIT cells, R848, or a combination of the two. Data in (D and E) are from two donors, both allogeneic to the DCs. Additional data related to this experiment are shown in Supplemental Fig 3.

Only MAIT cells induced 5-A-RU/MG–dependent DC maturation, because CD8^+^ CD161^+^ T cells and CD8^+^ CD161^−^ T cells sorted from the same donors were not activated by the vitamin B2 intermediate (Fig. 5A) and did not induce DC activation (Fig. 5B); however, as expected, CD8 cells were responsive to TLR-matured DCs (Fig. 5A).

### Freshly sorted human MAIT cells induce DC maturation

It has been shown that MAIT cell cytotoxicity can be enhanced upon in vitro culture, following upregulation of granzyme B and perforin (22). To exclude a possible effect of the in vitro culture on MAIT cell function, we investigated whether freshly sorted MAIT cells could also induce DC maturation. In line with recently published results (23), both fresh MAIT cells and MAIT cell lines homogeneously expressed CD27 and CD28, whereas they upregulated CD56 in culture (Fig. 5C). NKG2D expression varied the most across the donors tested, particularly upon culture (Fig. 5C). A small percentage of cells expressed NKG2A, whereas we did not observe any KIR2DL1 expression (Fig. 5C). Freshly sorted MAIT cells were activated by 5-A-RU/MG in a dose-dependent manner, and their activation was enhanced by TLR ligands (Fig. 5D, Supplemental Fig. 3A). Freshly sorted MAIT cells also induced DC maturation and, in the presence of low doses of 5-A-RU/MG, enhanced TLR-dependent DC activation, as shown by upregulation of surface markers (Fig. 5E) and secretion of IL-12p70 and IL-12p40 (Supplemental Fig. 3B, 3C).

### Maturation of primary DCs via MAIT cells leads to transactivation of NK cells

**FIGURE 6.**
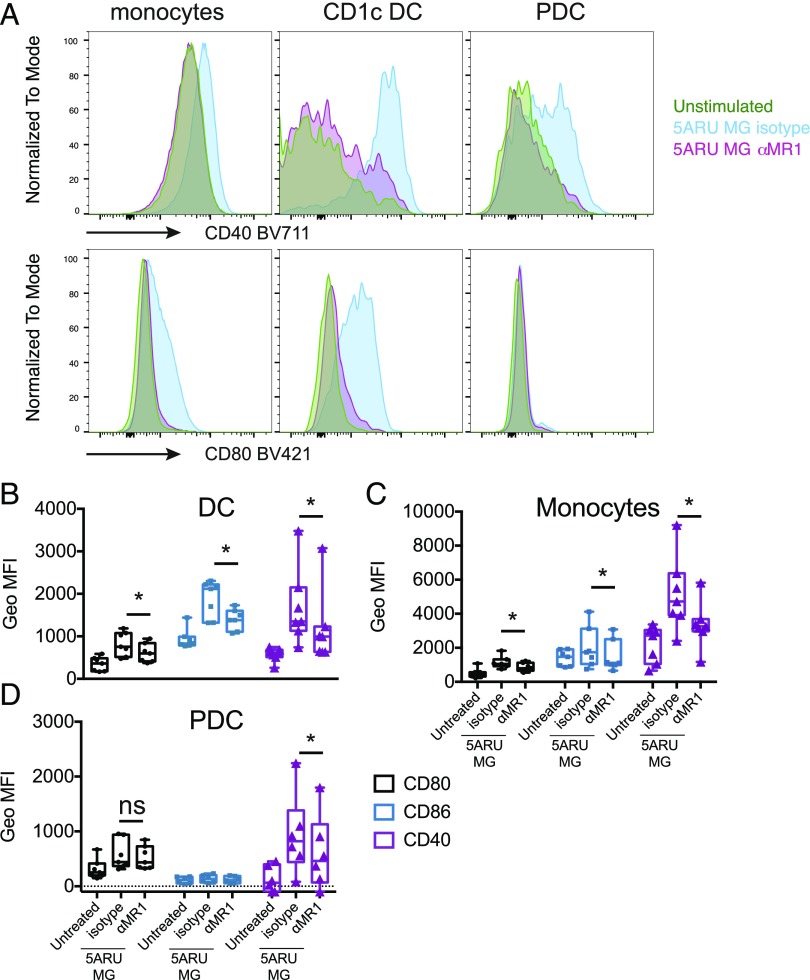
Human MAIT cells induce maturation of primary blood DCs. (**A**) Monocytes, CD1c DCs, and PDCs were gated as shown in Supplemental Fig. 4. Depicted is the cell surface expression of CD40 (upper panels) and CD80 (lower panels) in cells that were left unstimulated (green) or were stimulated for 16 h with 5-A-RU/MG, in the presence (pink) or absence (light blue) of anti-MR1 blocking Ab. (**B**–**D**) Cumulative data for seven donors (six for PDCs) depicting geometric mean fluorescence intensity (MFI) for CD80 (black), CD86 (blue), and CD40 (purple) for cells that were left unstimulated or stimulated with 5-A-RU/MG, in the presence or absence of anti-MR1 blocking Ab. Data are depicted as box-and-whisker plots, with all points indicated, ±SD. *p < 0.05.

**FIGURE 7.**
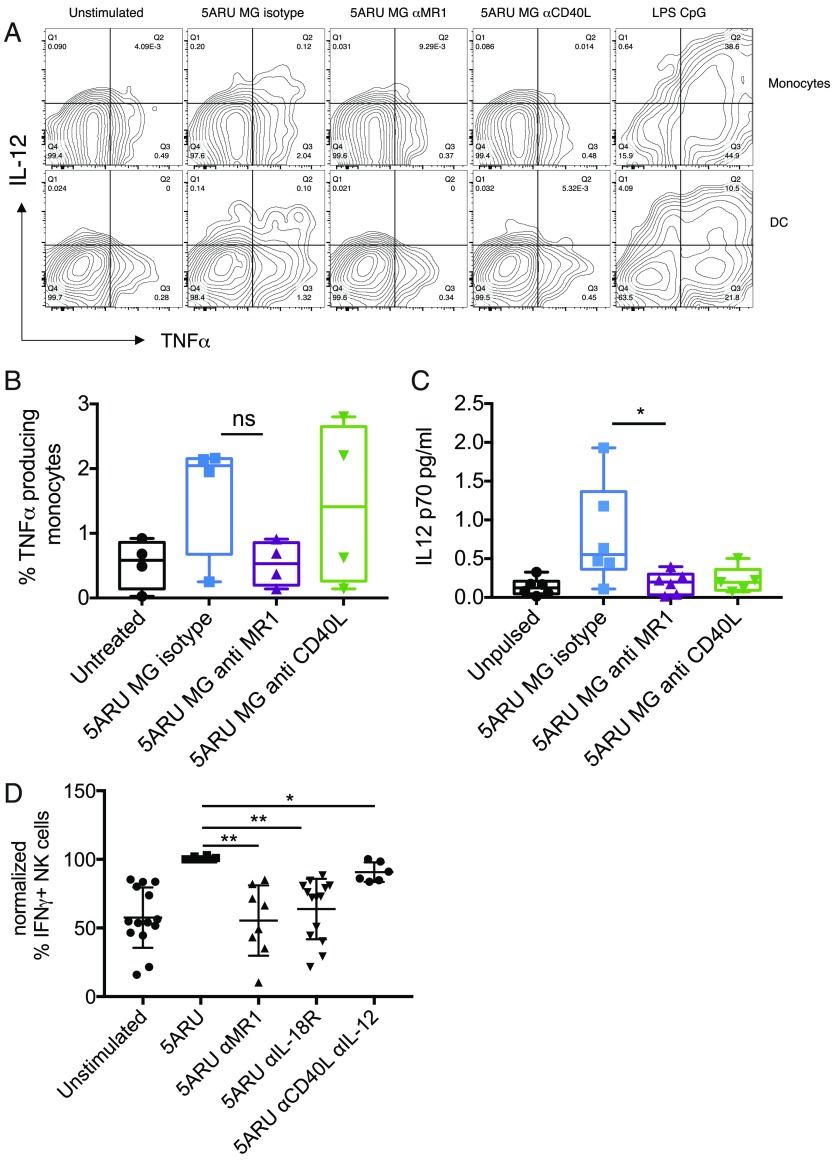
(**A**) FACS dot plots showing intracellular expression of IL-12 and TNF-α in monocytes and DCs (gated as in Fig. 6, Supplemental Fig. 4), 16 h after incubation with 5-A-RU/MG in the presence or absence of anti-MR1 or anti-CD40L blocking Abs. Profiles of LPS- and CpG-stimulated cells are shown as positive control. (**B**) Cumulative data for the intracellular TNF-α expression described in (A). Data are depicted as box-and-whisker plots, with all points indicated (*n* = 4). (**C**) IL-12p70 detected by Simoa immunoassay in whole blood stimulated for 16 h with 5-A-RU/MG in the presence or absence of anti-MR1 or anti-CD40L blocking Abs. Cumulative data (*n* = 6; *n* = 5 for anti-CD40L) are depicted as box-and-whisker plots, with all points indicated ±SD. (**D**) PBMCs were stimulated with 5-A-RU/MG in the presence or absence of blocking Abs to MR1 (*n* = 8), IL-18R (*n* = 14), and IL-12 and CD40L (*n* = 6), and the percentage of IFN-γ–secreting NK cells was determined by FACS after 16 h. The response with Ag was normalized to 100%; we observed background IFN-γ in the majority of the donors tested. Each symbol represents a donor. *p < 0.05, **p < 0.01.

To assess whether our results obtained with monocyte-derived DCs could be extended to primary cells, we investigated the immune-modulatory activity of MAIT cells ex vivo, in whole blood. Using an X50 BD Symphony five-laser flow cytometer, which allows simultaneous measurement of up to 30 parameters, we developed a gating strategy to identify myeloid subpopulations and T, B, and NK cells (Supplemental Fig. 4A). Freshly collected blood from healthy volunteers was incubated with 5-A-RU/MG in the presence or absence of anti-MR1 or anti-CD40L Abs, and cells were stained 16 h later. Activation of MAIT cells in response to 5-A-RU/MG stimulation was revealed by induction of TNF-α secretion, CD137 upregulation, and Vα7.2 TCR downregulation (Supplemental Fig. 4B). As expected, MAIT cell activation could be blocked by anti-MR1 treatment (Supplemental Fig. 4B). Upon 5-A-RU/MG exposure, monocytes, CD1c DCs, and plasmacytoid DCs (PDCs) upregulated CD40, CD86, and CD80, markers that are indicative of maturation. Significantly, blocking MAIT cell activation with anti-MR1 also abrogated this maturation process (Fig. 6). Furthermore, we detected intracellular IL-12p40 and TNF-α in activated monocytes and DCs (Fig. 7A, 7B). TNF-α production could be specifically blocked by treatment with anti-MR1 but not anti-CD40L (Fig. 7B). Using a highly sensitive Simoa immunoassay, we detected secretion of IL-12p70 in the above cocultures, which was also reduced by anti-MR1 and anti-CD40L (Fig. 7C). Finally, MAIT cell activation also led to NK cell transactivation in an MR1- and IL-18–dependent manner (Fig. 7D).

## Discussion

In this article we have demonstrated that human MAIT cells rapidly upregulate CD40L upon activation by the cognate Ag 5-A-RU/MG presented by the nonclassical class I molecule MR1. Furthermore, we have shown that MAIT cells are able to instruct DC maturation in a CD40L- and MR1-dependent manner, resulting in secretion of bioactive IL-12. Notably, we have demonstrated MAIT-dependent DC maturation with primary CD1c DCs and PDCs, as well as monocyte-derived DCs.

It is now well accepted that DC maturation is key to the successful induction of adaptive T cell responses. Immature DCs patrolling peripheral tissues are exposed to a variety of maturation signals (pathogen-associated molecular patterns or danger-associated molecular patterns) that, through specific sensors (pattern recognition receptors), initiate the activation/maturation process, leading to DC migration to the draining lymph node and Ag presentation to naive T cells (24). Pattern recognition receptors tailor the quality of the adaptive immune response to the nature of the pathogen (25). Full DC maturation, as well as generation of cytotoxic T cell responses, also requires engagement of the TNF superfamily receptor CD40 (15). In addition to rare Ag-specific activated CD4^+^ T cells (26), innate lymphocytes, such as NK cells, iNKT cells, and γδ T cells, have been shown to constitutively express CD40L and trigger CD40L-dependent DC maturation (17, 18, 27). We have now demonstrated that, upon Ag-specific activation, MAIT cells also rapidly upregulate CD40L at the cell surface and, in synergy with TLR ligands, induce secretion of bioactive IL-12 by DCs. Our results complement a previous report demonstrating the presence of soluble CD40L in the supernatant of human MAIT cells cultures activated by bacteria, although the functional relevance of this observation was not addressed at the time (28). Altogether, these results point to an important immune-regulatory function of MAIT cells, in agreement with their observed ability to induce GM-CSF–dependent differentiation of inflammatory monocytes into monocyte-derived DCs (6).

We have observed that MAIT cells induced partial DC activation, even in the absence of exogenous ligands. We refer to this as basal MAIT cell autoreactivity, and future work will be required to determine whether it is due to endogenous ligand or medium-derived vitamins or a combination of the two. Activation of murine MAIT hybridomas has been observed in response to MR1-overexpressing APCs (29–31); although this was considered evidence of self-ligand presentation, the ligand was not identified. Recently, a subset of autoreactive and folate-reactive cells, whose activity was strictly dependent on the TCRβ-chain, was identified within the conventional Vα7.2-bearing MAIT population (32). We have also observed variability in the extent of basal autoreactivity within our Vα7.2^+^ CD161^+^ MAIT sorted cells, which could be accounted for by a heterogeneous TCRβ repertoire within our blood donor cohort (M. Salio, unpublished observations). Although we have observed basal autoreactivity in autologous and allogeneic settings, further experiments are warranted to investigate whether, despite the monomorphic nature of MR1, basal autoreactivity of MAIT cells may be modulated by differential expression of KIR or by HLA mismatch between MAIT cells and DCs.

Finally, in addition to DC maturation, MAIT cell activation in whole blood led to NK cell transactivation in an MR1- and IL-18–dependent manner. NK cell transactivation has also been observed upon iNKT cell activation with a number of iNKT cell agonists, such as α-GalCer (33) and its nonglycosidic derivatives (34), and it is essential for the antimetastatic activity of iNKT cells and the sustained IFN-γ secretion observed upon iNKT cell activation (33). The in vivo consequences of MAIT cell activation upon 5-A-RU/MG triggering will be the subject of future studies.

Similarities and differences have emerged between the two populations of innate-like T cells for which synthetic Ags and tetramers are available: MAIT and iNKT cells. Parallel thymic selection processes on double-positive thymocytes lead to the appearance of cells that need further shaping in the periphery, in a process that is dependent on microbial flora (35–37). Although a shared developmental niche has been suggested to regulate iNKT cell numbers (35), it is unclear to what extent MAIT and iNKT cell function is coregulated in the periphery. Nevertheless, the ability of MAIT and iNKT cells to regulate DC function (this article) and differentiation (6) warrants further investigation. iNKT cell agonists are currently being tried in the clinic to enhance Ag-specific immune responses (38–40), and we anticipate that harnessing MAIT cells in humans may offer additional important strategies to promote the development of mucosal and systemic immunity. Indeed, the higher precursor frequency and memory phenotype of MAIT cells (41) render them particularly attractive “natural adjuvants” to enhance immune responses against pathogens and for cancer immunotherapy (42).

## Supplementary Material

Data Supplement
