# Supplementary material for: Activation of Human Mucosal-Associated Invariant T Cells Induces CD40L-Dependent Maturation of Monocyte-Derived and Primary Dendritic Cells
Source: J Immunol. 2017 Sep 6;199(8):2631–8. doi: 10.4049/jimmunol.1700615 (PMC5632842; doi:10.4049/jimmunol.1700615)
Supplement: Data Supplement [file JI_1700615.zip › JI_1700615_Supplemental_Figures_1.pdf]

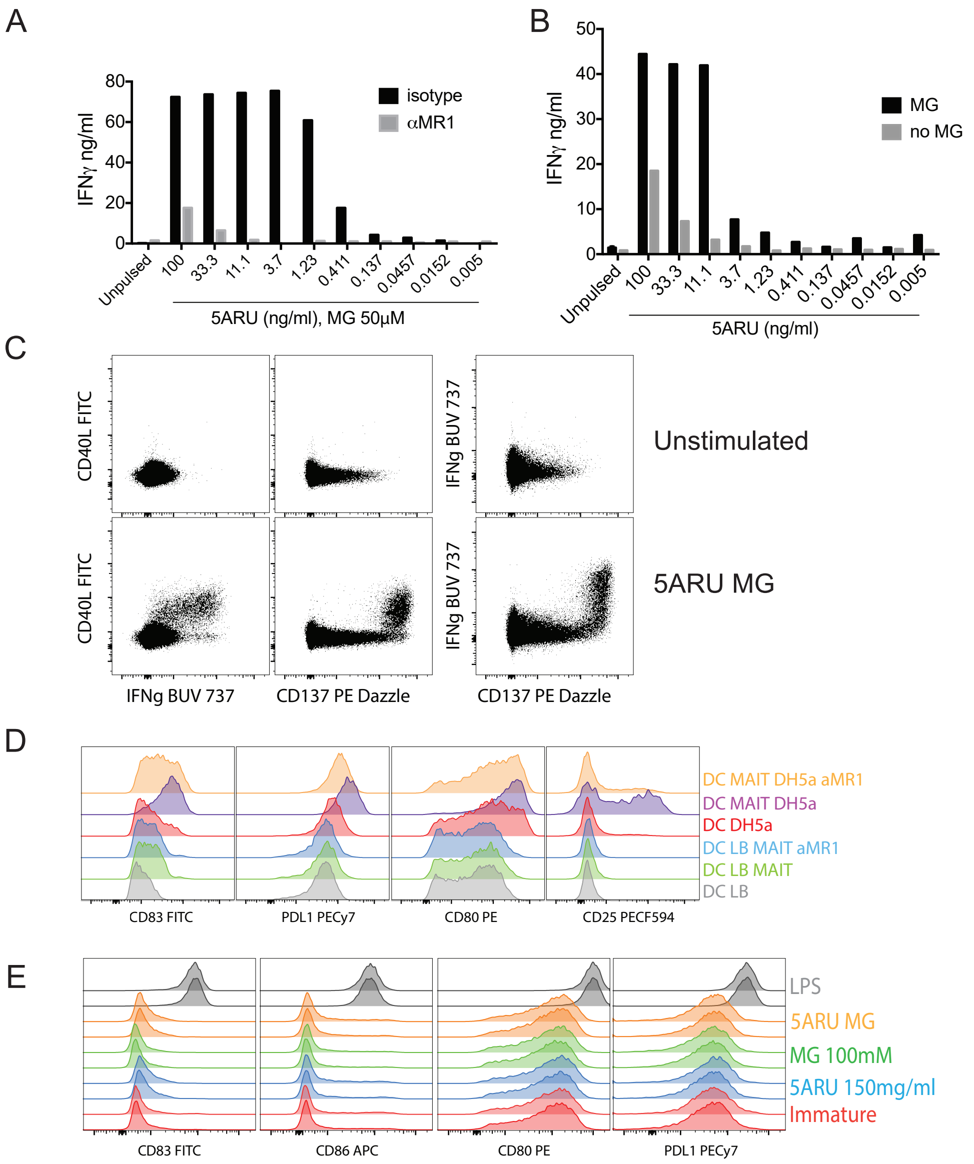

Supplemental Figure 1: Characterization of 5-A-RU. A) Monocyte derived DC were pulsed with the indicated concentrations of 5-A-RU and constant methyl glyoxal (MG) and co-incubated with MAIT cells in the presence of the blocking anti-MR1 antibody or a relevant isotype control. B) 5-A-RU was titrated as above, in the presence or absence of exogenous MG. IFN-g secretion by MAIT cells was measured in the supernatants after 36 hours. One representative experiment of three.

C) MAIT cell activation leads to CD40L upregulation. This panel complements Fig 1. Dot plots depict CD137 and IFN-γ expression in CD40L positive cells, and IFN-γ expression in CD137 positive cells. One representative experiment of three.

D) MAIT cells induce MR1-dependent DC maturation in response to bacterial supernatant. Immature DC were incubated with LB or DH5-α supernatant in the presence or absence of allogeneic MAIT cells and anti-MR1 antibodies. Depicted are expression levels of CD83, PDL1, CD80 and CD25 as detected by flow cytometry after 36 hours. One representative experiment of three.

E) 5-A-RU or methyl glyoxal do not induce DC maturation in the absence of MAIT cells. Immature DC (red histograms) were incubated with the indicated concentrations of 5-A-RU and/or MG or LPS in the absence of MAIT cells. Depicted are expression levels of CD83, CD86, CD80 and PDL1 as detected by flow cytometry after 36 hours. Staining profile of duplicate wells is shown. One representative experiment of three. Differences in DC FACS profiles between figures 2 and S2 are attributable to variations amongst healthy donors, likely due to differences in basal autoreactivity, influenced by the TCRb repertoire of the MAIT cells.

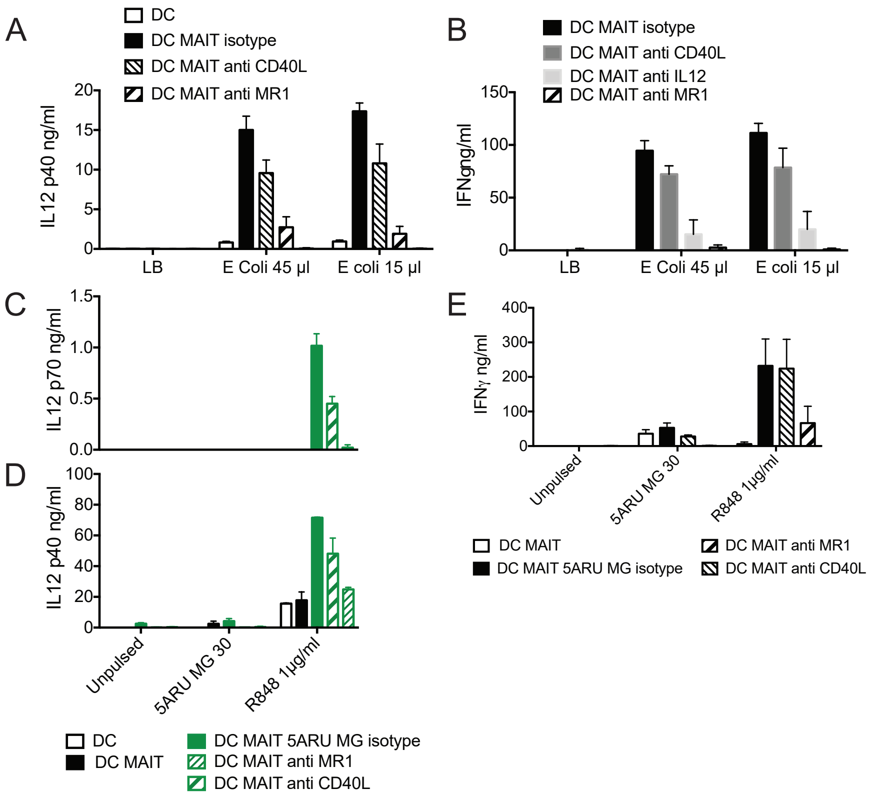

Supplemental figure 2 (complements Fig 4): Synergy between human MAIT cell agonists and TLR agonists. A) IL-12p40 and (B) IFN-γ levels in the supernatant of DC pulsed with E. coli supernatant in the presence or absence of MAIT cells and blocking anti-CD40L, anti IL-12 or anti-MR1 antibodies.
IL-12p70 (C), IL-12p40 (D) and IFN-γ levels (E) in the supernatant of DC pulsed with the indicated concentrations (ng/ml) of 5-A-RU/MG or R848 (µg/ml) in the presence or absence of MAIT cells and blocking anti-CD40L or anti-MR1 antibodies. Data are from two donors, one autologous and one allogeneic to the DC; values represent mean+/- SD.

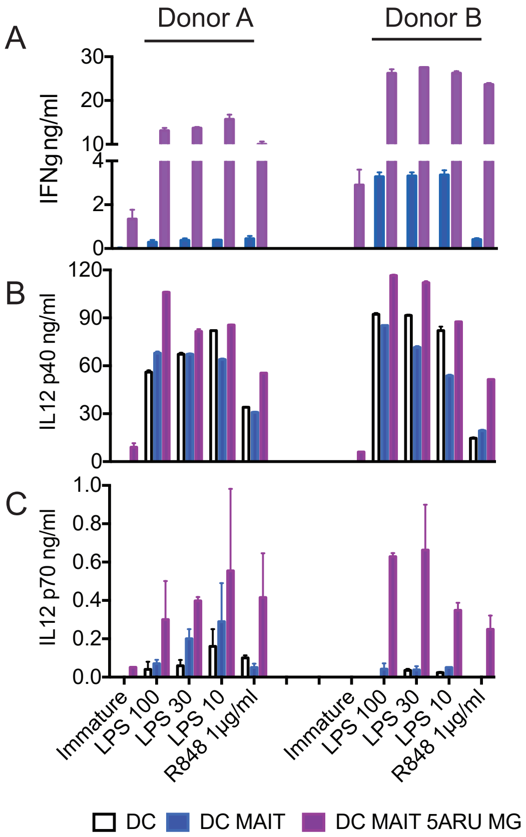

Supplemental Figure 3: Freshly sorted MAIT cells induce DC maturation. MAIT cells were freshly sorted from two donors and incubated with allogeneic DC in the presence of the indicated concentrations of TLR ligands (ng/ml for LPS, mg/ml for R848), with or withour 30ng/ml 5-A-RU/MG. Plotted are the concentrations (ng/ml, mean +/- SD) of IFN-g (A), IL-12p40 (B) and IL-12p70 (C) in the supernatants harvested after 36 hours. This figure complements Fig. 5 C-E.

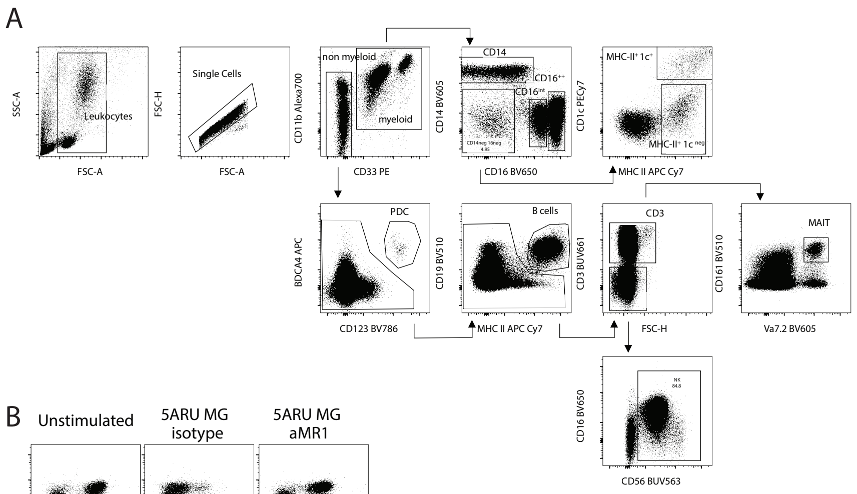

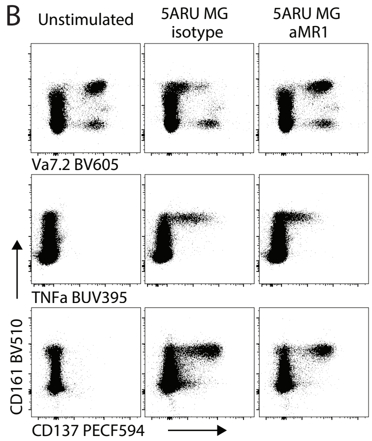

Supplemental Figure 4: Human MAIT cells induce maturation of primary blood DC. A) Gating strategy for the experiment shown in panel B and in Figures 6 and 7. The main myeloid and non-myeloid populations are shown in the panels. B) MR1 dependent MAIT cell activation in whole blood pulsed 16 hours with 5-A-RU/MG with or without blocking anti-MR1 antibody. Expression of Vα7.2, TNF-α and CD137 on CD161 bright cells is depicted. One experiment representative of five, for cumulative data on anti-MR1 blocking see Fig. 1B.
